# Educational

**Published:** 1877-10

**Authors:** 


### EDUCATIONAL.

The various dental colleges of this country are about entering upon their annual sessions, and from reports thus far, the indications are that the attendance will be good--probably better than usual. An increase in members is not more desirable than an improvement in the preliminary preparation of those who do enter, though doubtless progress in being made in this respect, not as much however, as we could wish. It is a matter of regret that the occasion exists for the remark, "I will go to such an institution because there is, there, no preliminary examination as to previous education and training."

As to how much ground there is for statements of this character we are not advised, and will not venture an opinion; but that there is any ground for it anywhere, is exceedingly unfortunate for the profession.

When the entrance door to our colleges is wide and of easy passage, the graduation door will be about as wide and easy.

There should be, in the dental colleges in this country, some uniform rules and regulations in reference to the admission of students, in respect to literary and scientific attainments, and the attainments for graduation, etc.

The members of the profession are, to a large extent, responsible for the want of preparation of those who seek to enter our dental colleges. Students who are totally unprepared are too often encouraged to enter our colleges, and as a result, labor under great embarrassment all the way through; and if graduated at all, it is by pressing and urging out an admissable examination.

In order to make the course of professional study and training most efficient, the student should possess the habit of studying, and a good knowledge of language; not only of the English, but of those languages that enter so largely into medical literature, and a good knowledge of the common sciences would be of very great advantage. By too many of our students, and unfortunately by some who were students a good while ago, these things are lightly esteemed. May the time soon come when it will be quite otherwise.

